# Context dependency of maintenance communities of invasive parasites under climate change: a case study of mussels and intestinal copepods in the Wadden Sea

**DOI:** 10.1098/rsif.2025.0370

**Published:** 2025-11-05

**Authors:** E. Rosa Jolma, Anieke van Leeuwen, K. Mathias Wegner, David W. Thieltges, J. A. P. (Hans) Heesterbeek, Mick G. Roberts

**Affiliations:** ^1^ Department of Coastal Systems, NIOZ Royal Netherlands Institute for Sea Research, Texel, The Netherlands; ^2^ Faculty of Veterinary Medicine, Utrecht University, Utrecht, The Netherlands; ^3^ Alfred Wegener Institute, Wadden Sea Station Sylt, List, Germany; ^4^ Groningen Institute for Evolutionary Life Sciences (GELIFES), University of Groningen, Groningen, The Netherlands; ^5^ New Zealand Institute for Advanced Study, Massey University, Auckland, New Zealand

**Keywords:** climate change, ecological modelling, *Mytilicola*, *Mytilus edulis*, parasite dynamics, population dynamics

## Abstract

Climate change can impact the persistence of native and invasive parasites and their effects on hosts. Given the complexity of interactions in natural systems, models based on parasite–host systems can be helpful to explore long-term impacts. We investigate how two intestinal parasitic copepods impact host populations, and how the predicted temperature increase by year 2100 may affect the persistence and impacts of the parasites. We study *Mytilicola intestinalis* (a specialist established in blue mussels, *Mytilus edulis*) and *Mytilicola orientalis* (a recent invader infecting mussels and Pacific oysters, *Magallana gigas*) in the Wadden Sea. The parasites are non-lethal but can influence host maturation and fecundity. Using a mathematical model parametrized with empirical, field and literature data, we explore how temperature increase affects parasite basic reproduction numbers and the long-term population trends of parasites and mussels. Temperature increase reduces mussel populations below the critical community size for *M. intestinalis* persistence, while allowing *M. orientalis* to persist without oysters. *M. orientalis* does not have a negative effect on the host population in additional to that of *M. intestinalis* when both are present. We show that environmental change can have qualitatively different effects on related parasites by changing the role of the shared host as a maintenance population.

## 1. Introduction

The ability of a parasite to persist in an ecosystem or to successfully invade new ecosystems depends on the availability of suitable host species and will further depend on the specific environmental context rather than being a fixed characteristic of the host–parasite system [1]. Factors influencing the ultimate outcome include environmental changes that may alter the species composition and population densities of host communities as well as the life cycles of the parasites directly [2,3]. Furthermore, since parasites can affect host populations [4–6], the presence of multiple parasite species may also affect the persistence of parasite species sharing a host species [7]. We will use the term persistence, but also refer to maintenance hosts, maintenance communities and reservoirs as defined in [8] within the boundaries of our study system, in the knowledge that the use of this terminology is ecosystem and context specific [1].

One parasite trait that can influence how environmental change affects their persistence is host specificity. Specialist parasites are dependent on the success of their only host species, while generalists can thrive even if one host species is lost altogether [2]. Other relevant parasite traits are their pre-existing environmental adaptations and the genetic diversity of the population, as a higher variation in the genetic pool increases adaptive potential [2,3]. All of these traits can differ between native and introduced species: typical successful invaders are more generalist on the specialist–generalist continuum [9,10] and can have pre-existing environmental adaptations that do not completely align with the conditions of the introduced range, while species often go through genetic bottlenecks on their invasion pathway, resulting in reduced genetic diversity [3,10]. Introduced parasites can also have pre-existing traits that allow them to better adapt to future changing conditions in their new environment. However, the presence of other parasites and hosts in the system can affect the invasion success of novel parasites, both through direct interactions and through their effects on host populations [2,11]. To predict the effect of environmental changes on parasite invasion success, persistence, dynamics and host population interactions, one thus needs to incorporate the complexity of other relevant hosts and parasites in a specific system. Our current understanding of the interplay of environmental shifts such as climate change and parasite invasions is limited and dependent on the parasite–host system investigated [12]. Given the challenging complexity of natural systems, models based on well-studied parasite–host systems can be helpful to explore the long-term impacts of environmental changes on parasite invasions.

In this study, we investigated the effect of environmental change in the form of increasing temperature on a community consisting of two species of intestinal parasitic copepods with simple life cycles and two partially shared ectothermic host species in a temperate coastal ecosystem. This parasite–host system is particularly suited to investigate climate change effects on parasite invasions as a wealth of observational and experimental data, including on temperature effects, exists from the study area. In addition, the Wadden Sea is subjected to significant warming. The mean annual water temperature was approximately $10^{\circ }\text{C}$ before climate change impacts and is predicted to increase to 14–$15^{\circ }\text{C}$ by the end of the century [13,14]. The parasitic copepod, *Mytilicola intestinalis,* is a specialist that only infects native blue mussels (*Mytilus edulis*) and arrived in the system approximately 90 years ago [15], hence it can be considered to be an established species. *Mytilicola intestinalis* was first detected in the Mediterranean in 1902, but the low genetic diversity in its invaded and putative native region may indicate that it has experienced a genetic bottleneck prior to its arrival in the North Sea [15]. A second congener, *Mytilicola orientalis,* arrived only 20 years ago with its original host, the invasive Pacific oyster (*Magallana/Crassostrea gigas*) from the western Pacific Ocean [15]. The genetic diversity of *M. orientalis* is high even in the invaded range due to hitchhiking on numerous oyster transports from the native range [15]. It is unclear if oysters are required for the persistence of *M. orientalis* in the invaded range. What is currently known is that the oysters and parasites arrived together and coexist throughout the invaded range. In the Baltic Sea, where *M. orientalis* was first detected in 2018, this coincided with the first successful spatfall of oysters [16,17], suggesting that oysters need to be present for *M. orientalis* to establish. Neither parasite species increases host mortality within a temperature range of 10–$26^{\circ }\text{C}$, but *M. orientalis* causes a slight reduction in mussel shell growth and *M. intestinalis* a reduction in mussel condition [14,18]. Direct competition between the parasite species does not affect infection rates [19]. An increase in temperature accelerates the development of both parasites and also shortens the lifespan of infectious free-living juvenile stages in most of the temperature range [14]. For *M. intestinalis,* the decrease in juvenile lifespan occurs across the whole range of temperatures, but for *M. orientalis* juveniles, the longest lifespan occurs at $14^{\circ }\text{C}$ instead of $10^{\circ }\text{C}$ before a decline with increasing temperature [14], as a possible sign of inadequate adaptation for current winter temperatures in the Wadden Sea, but simultaneously perhaps aligning better with the predicted future temperature range.

Given the current presence of both parasites in the Wadden Sea, we investigated three questions with the aid of a mathematical model, parametrized with our empirical, field and literature data:

(a) What conditions were required for *M. orientalis* to invade the system already occupied by *M. intestinalis*, and are oysters required for the persistence of *M. orientalis* in the community?
(b) Did the addition of the second invasive parasite increase the parasite impact on mussel populations?
(c) How is the predicted increase in temperature by year 2100 likely to affect the maintenance communities of the two parasites and their impact on blue mussel populations?

## 2. Methods

**Figure 1.**
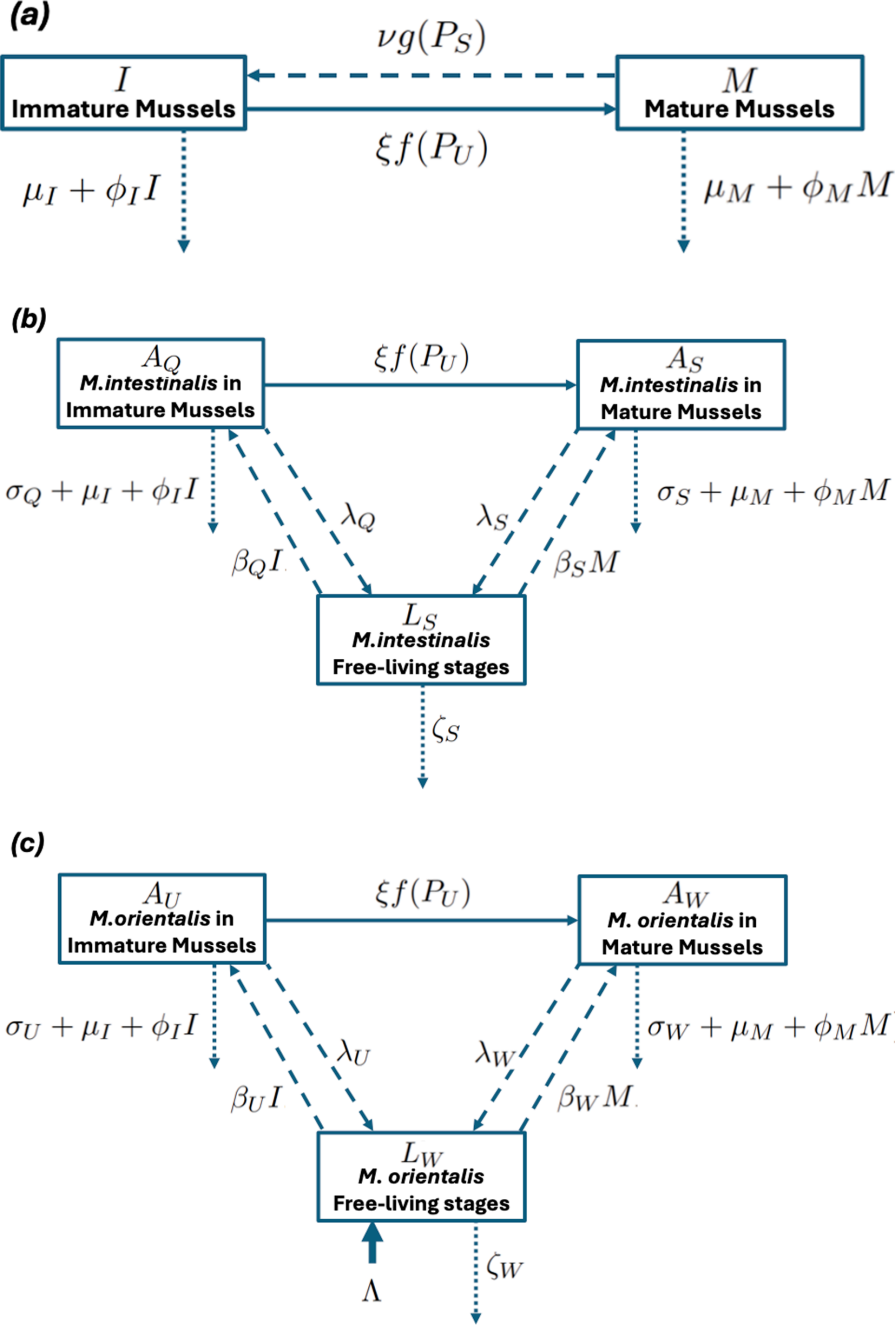
Structure of the models of the life cycles of (*a*) mussels, (*b*) *M. intestinalis* and (*c*) *M. orientalis*. Solid lines indicate maturation of hosts and input of free-living stages, dashed lines indicate reproduction of mussels and parasite stage transitions, dotted lines indicate loss of mussels or parasites.

The dynamics of the mussel (host) and parasite populations were modelled using a system of ordinary differential equations [20]. In our model, the host population consists of two simplified life stages: immature mussels that grow (I), and mature mussels that reproduce (M). Both stages can get infected with both parasites. Populations of both parasites consist of parasitic adult life stages that live in the intestine of hosts and infectious free-living juvenile stages that hatch from the eggs of the sexually reproducing adult parasites and spread to new hosts via seawater (see figure 1). Steady-state (equilibrium) values of the parasite populations were found using MatLab [21] as functions of the effect on host maturation due to infection with *M. orientalis* and the rate of immigration of *M. orientalis* from adjacent oyster populations. The steady states where just one parasite species is present were found using the fzero function; for both species present, fsolve was used. A steady state of *M. intestinalis* only exists when there is no immigration of *M. orientalis*. When there is immigration of *M. orientalis,* then steady states of that parasite and of both parasites together were computed. Stability of the steady states was checked by computing the eigenvalues of the appropriate Jacobian matrices. In addition, the differential equations were solved numerically over time using the ode45 function of MatLab [21], confirming the steady-state values and their stability. The results are presented as mean numbers of *M. intestinalis* in immature and mature hosts, $P_{Q}$ and $P_{S}$, respectively, and mean numbers of *M. orientalis* in immature and mature hosts, $P_{U}$ and $P_{W}$, respectively. Steady states in the absence of the other parasite are indicated with a # superscript, and in the presence of the other parasite are indicated with a ⋆ superscript.

### 2.1. The model

Let the population densities of immature and mature mussels be I and M, respectively. Assume that each subpopulation exhibits logistic dynamics, but without competition for space or resources between the immature and mature populations, and that competition for resources within each subpopulation increases mortality. Let the rate at which uninfected mature mussels produce immature offspring be ν, the rate at which uninfected mussels mature be ξ and the rates of mortality of immature and mature mussels be $\mu_{I}+\phi_{I}I$ and $\mu_{M}+\phi_{M}M$, respectively. The parameter ν depends on the local predator population, and ξ depend on the sea temperature and food availability. The population dynamics of the mussel population are described by the differential equations


(2.1) $$\begin{array}{rl} \frac{dI}{\;dt} & =\nu g\left(P_{S}\right)M-\left(\mu_{I}+\phi_{I}I+\xi f\left(P_{U}\right)\right)I\\ \frac{\;dM}{\;dt} & =\xi f\left(P_{U}\right)I-\left(\mu_{M}+\phi_{M}M\right)M, \end{array}$$

where $P_{S}$ is the mean number of *M. intestinalis* per mature mussel and $P_{U}$ is the mean number of *M. orientalis* per immature mussel. The functions f and g quantify a negative effect of the within-host parasite load on the maturation rate of immature hosts and offspring production per mature host, respectively. We model these as functions of the mean parasite loads in the two mussel life stages. As infection with *M. orientalis* delays maturity, we have f(0)=1 and $f^{\prime }(P_{U})<0$. Similarly, as infection with *M. intestinalis* reduces fecundity, we need g(0)=1 and $g^{\prime }(P_{S})<0$. Specific functional forms for f and g were determined related to observed parasite load distributions (see §2.2).

Setting $P_{S}=P_{U}=0$ for now, the steady-state (equilibrium) solutions of equation (2.1) satisfy $\xi I=\left(\mu_{M}+\phi_{M}M\right)M$ and $\nu M=\left(\mu_{I}+\phi_{I}I+\xi \right)I$. Let these solutions be $I_{0}$ and $M_{0}$. Hence, $M_{0}$ solves the cubic equation


$$F(M)=\phi_{I}M{\left(\phi_{M}M+\mu_{M}\right)}^{2}+\xi \left(\mu_{I}+\xi \right)\left(\phi_{M}M+\mu_{M}\right)-\nu \xi^{2}=0.$$

We have $F(0)=\mu_{M}\xi \left(\mu_{I}+\xi \right)-\nu \xi^{2}$ and $F^{\prime }(M)>0$ for M>0. Hence, there is a unique solution $M_{0}>0$ if and only if $\nu \xi >\mu_{M}\left(\mu_{I}+\xi \right)$. The parameter values used for modelling the mussel population are summarized in table 1.

**Table 1.** Mussel parameter values used in the model.

at $10^{\circ }\text{C}$	
birth rate of hosts	$\nu =0.03\;{\text{d}}^{-1}$
minimum death rate of immature hosts	$\mu_{I}=4.0\times 10^{-3}\;{\text{d}}^{-1}$
minimum death rate of mature hosts	$\mu_{M}=2.0\times 10^{-3}\;{\text{d}}^{-1}$

at $15^{\circ }\text{C}$	
birth rate of hosts	$\nu =0.02\;{\text{d}}^{-1}$
minimum death rate of immature hosts	$\mu_{I}=4.4\times 10^{-3}\;{\text{d}}^{-1}$
minimum death rate of mature hosts	$\mu_{M}=2.2\times 10^{-3}\;{\text{d}}^{-1}$

at both temperatures	
competition coefficient immature hosts	$\phi_{I}=1.8\times 10^{-4}\;{\text{m.}}^{-1}\;{\text{d}}^{-1}$
competition coefficient mature hosts	$\phi_{M}=3.6\times 10^{-4}\;{\text{m.}}^{-1}\;{\text{d}}^{-1}$
maturation rate immature to mature	$\xi =(1.4,3.0)\times 10^{-3}\;{\text{d}}^{-1}$ variable

m. signifies one individual mussel (immature or mature) per unit volume; d signifies one day.

Let the total number of *M. intestinalis* in the immature and mature mussel populations be $A_{Q}=P_{Q}I$ and $A_{S}=P_{S}M$, respectively, and the density of parasite-free-living stages in the water be $L_{S}$. Hence, the dynamics of the parasite population may be modelled by the differential equations


$$\begin{array}{rl} & \frac{dA_{Q}}{\;dt}=\beta_{Q}IL_{S}-\left(\sigma_{Q}+\mu_{I}+\phi_{I}I+\xi f\left(P_{U}\right)\right)A_{Q}\\ & \frac{\;dA_{S}}{\;dt}=\beta_{S}ML_{S}+\xi f\left(P_{U}\right)A_{Q}-\left(\sigma_{S}+\mu_{M}+\phi_{M}M\right)A_{S}\\ & \frac{\;dL_{S}}{\;dt}=\lambda_{Q}A_{Q}+\lambda_{S}A_{S}-\zeta_{S}L_{S}. \end{array}$$

The parameter values used for the *M. intestinalis* population are summarized in table 2.

**Table 2.** *Mytilicola intestinalis* parameter values used in the model.

at $10^{\circ }\text{C}$	
transmission rate to immature hosts	$\beta_{Q}=2.2\times 10^{-4}\;\text{p.}\;\ell .^{-1}\;{\text{m.}}^{-1}\;{\text{d}}^{-1}$
transmission rate to mature hosts	$\beta_{S}=4.6\times 10^{-4}\;\text{p.}\;\ell .^{-1}\;{\text{m.}}^{-1}\;{\text{d}}^{-1}$
parasite mortality in immature hosts	$\sigma_{Q}=7.8\times 10^{-3}\;{\text{d}}^{-1}$
parasite mortality in mature hosts	$\sigma_{S}=7.8\times 10^{-3}\;{\text{d}}^{-1}$
parasite transmission from immature hosts	$\lambda_{Q}=0.38\;\ell .\;{\text{p.}}^{-1}\;{\text{d}}^{-1}$
parasite transmission from mature hosts	$\lambda_{S}=0.38\;\ell .\;{\text{p.}}^{-1}\;{\text{d}}^{-1}$
loss rate of free-living stages	$\zeta_{S}=0.29\;{\text{d}}^{-1}$

at $15^{\circ }\text{C}$	
transmission rate to immature hosts	$\beta_{Q}=2.8\times 10^{-4}\;\text{p.}\;\ell .^{-1}\;{\text{m.}}^{-1}\;{\text{d}}^{-1}$
transmission rate to mature hosts	$\beta_{S}=5.4\times 10^{-4}\;\text{p.}\;\ell .^{-1}\;{\text{m.}}^{-1}\;{\text{d}}^{-1}$
parasite mortality in immature hosts	$\sigma_{Q}=0.011\;{\text{d}}^{-1}$
parasite mortality in mature hosts	$\sigma_{S}=0.011\;{\text{d}}^{-1}$
parasite transmission from immature hosts	$\lambda_{Q}=0.53\;\ell .\;{\text{p.}}^{-1}\;{\text{d}}^{-1}$
parasite transmission from mature hosts	$\lambda_{S}=0.53\;\ell .\;{\text{p.}}^{-1}\;{\text{d}}^{-1}$
loss rate of free-living stages	$\zeta_{S}=0.4\;{\text{d}}^{-1}$

at both temperatures	
parasite effect on host fecundity	$g(P_{S})=1/\left(1+0.1\;\log(1+P_{S})\right)$

m. signifies one individual mussel (immature or mature) per unit volume; p. signifies one parasite; ℓ signifies one unit of free-living stages per unit volume; d signifies one day.

The next-generation matrix for *M. intestinalis* is


$$K_{in}=\left(\begin{array}{ccc} 0 & 0 & \beta_{Q}I_{0}\zeta_{S}^{-1}\\ 0 & 0 & \beta_{Q}I_{0}\zeta_{S}^{-1}\xi T_{Q}+\beta_{S}M_{0}\zeta_{S}^{-1}\\ \lambda_{Q}T_{Q} & \lambda_{S}T_{S} & 0 \end{array}\right),$$

where $T_{Q}={\left(\sigma_{Q}+\mu_{I}+\phi_{I}I_{0}+\xi \right)}^{-1}$ and $T_{S}={\left(\sigma_{S}+\mu_{M}+\phi_{M}M_{0}\right)}^{-1}$ (see Diekmann *et al.* [20] chapter 11). The basic reproduction number ($R_{0}$) for *M. intestinalis* is the largest eigenvalue of $K_{in}$,


$$R_{in}=\sqrt{\lambda_{Q}T_{Q}\beta_{Q}I_{0}\zeta_{S}^{-1}+\lambda_{S}T_{S}\beta_{S}M_{0}\zeta_{S}^{-1}+\xi T_{Q}\lambda_{S}T_{S}\beta_{Q}I_{0}\zeta_{S}^{-1}}.$$

The first term under the square root represents that a parasite in an immature mussel produces free-living stages at the rate $\lambda_{Q}$ for time $T_{Q}$, and that free-living stages infect immature mussels at the rate $\beta_{Q}I_{0}$ for time $\zeta_{S}^{-1}$. Hence, it represents the expected number of parasites produced in immature mussels by a single parasite in an immature mussel, without the involvement of mature mussels. Similarly, the second term represents the expected number of parasites produced in mature mussels by a single parasite in a mature mussel, without the involvement of immature mussels. The third term is the proportion of parasites in immature mussels that are still infecting the mussel after it has matured, $\xi T_{Q}$, multiplied by the rate at which parasites in mature mussels produce free-living stages and the time for which they do that, $\lambda_{S}T_{S}$, multiplied by the rate at which free-living stages infect immature mussels and the time for which they do that, $\beta_{Q}I_{0}\zeta_{S}^{-1}$. Hence, it represents the expected number of parasites produced in immature mussels by a single parasite in an immature mussel, following the maturity of the host mussel. Each of these ingredients is added to produce the expected number of secondary parasites due to a primary parasite. The square root commonly arises when characterizing $R_{0}$ for a parasite with a two-stage life cycle [20]. This is because $R_{0}$ describes growth/decline in terms of subsequent generations of infected individuals. If a system has different host stages or species that alternate in generations, such as here and for many host–vector systems, one has to look two generations ahead to characterize growth in the same stage/species. Per-generation growth, hence, comes with a square root. If $R_{in}<1$ the parasite population cannot persist in the host population, and if $R_{in}>1$ the parasite can invade and persist [20].

Similarly, let the total number of *M. orientalis* in the immature and mature mussel populations be $A_{U}=P_{U}I$ and $A_{W}=P_{W}M$, respectively, and the density of parasite-free-living stages in the water be $L_{W}$.

The dynamics of the parasite populations are described by


$$\begin{array}{rl} \frac{dA_{U}}{\;dt} & =\beta_{U}IL_{W}-\left(\sigma_{U}+\mu_{I}+\phi_{I}I+\xi f\left(P_{U}\right)\right)A_{U}\\ \frac{\;dA_{W}}{\;dt} & =\beta_{W}ML_{W}+\xi f\left(P_{U}\right)A_{U}-\left(\sigma_{W}+\mu_{M}+\phi_{M}M\right)A_{W}\\ \frac{\;dL_{W}}{\;dt} & =\Lambda +\lambda_{U}A_{U}+\lambda_{W}A_{W}-\zeta_{W}L_{W}. \end{array}$$

The term Λ accounts for immigration of *M. orientalis* from adjacent oyster host populations. As our focus is on the population dynamics of mussels and their parasites, we do not explicitly model *M. orientalis* in oyster populations. We assume that mussels are infected by *M. orientalis* free-living stages generated by infected mussels and oysters and present results over a range of Λ values reflecting different population densities of oysters and *M. orientalis* adjacent to mussel populations. The parameter values used for the *M. orientalis* population are summarized in table 3.

**Table 3.** *Mytilicola orientalis* parameter values used in the model.

at $10^{\circ }\text{C}$	
transmission rate to immature hosts	$\beta_{U}=1.7\times 10^{-4}\;\text{p.}\;{\ell .}^{-1}\;{\text{m.}}^{-1}\;{\text{d}}^{-1}$
transmission rate to mature hosts	$\beta_{W}=3.7\times 10^{-4}\;\text{p.}\;\ell .^{-1}\;{\text{m.}}^{-1}\;{\text{d}}^{-1}$
parasite mortality in immature hosts	$\sigma_{U}=7.8\times 10^{-3}\;{\text{d}}^{-1}$
parasite mortality in mature hosts	$\sigma_{W}=7.8\times 10^{-3}\;{\text{d}}^{-1}$
parasite transmission from immature hosts	$\lambda_{U}=0.41\;\ell .\;{\text{p.}}^{-1}\;{\text{d}}^{-1}$
parasite transmission from mature hosts	$\lambda_{W}=0.41\;\ell .\;{\text{p.}}^{-1}\;{\text{d}}^{-1}$
loss rate of free-living stages	$\zeta_{W}=0.25\;{\text{d}}^{-1}$

at $15^{\circ }\text{C}$	
transmission rate to immature hosts	$\beta_{U}=2.2\times 10^{-4}\;\text{p.}\;\ell .^{-1}\;{\text{m.}}^{-1}\;{\text{d}}^{-1}$
transmission rate to mature hosts	$\beta_{W}=4.4\times 10^{-4}\;\text{p.}\;\ell .^{-1}\;{\text{m.}}^{-1}\;{\text{d}}^{-1}$
parasite mortality in immature hosts	$\sigma_{U}=9.8\times 10^{-3}\;{\text{d}}^{-1}$
parasite mortality in mature hosts	$\sigma_{W}=9.8\times 10^{-3}\;{\text{d}}^{-1}$
parasite transmission from immature hosts	$\lambda_{U}=0.7\;\ell .\;{\text{p.}}^{-1}\;{\text{d}}^{-1}$
parasite transmission from mature hosts	$\lambda_{W}=0.7\;\ell .\;{\text{p.}}^{-1}\;{\text{d}}^{-1}$
loss rate of free-living stages	$\zeta_{W}=0.3\;{\text{d}}^{-1}$

at both temperatures	
parasite effect on maturation rate	$f(P_{U})=1/\left(1+0.2\;\log(1+P_{U})\right)$

m. signifies one individual mussel (immature or mature) per unit volume; p. signifies one parasite; ℓ signifies one unit of free-living stages per unit volume; d signifies one day.

The basic reproduction number for *M. orientalis* is


$$R_{or}=\sqrt{\lambda_{U}T_{U}\beta_{U}I_{0}\zeta_{W}^{-1}+\lambda_{W}T_{W}\beta_{W}M_{0}\zeta_{W}^{-1}+\xi T_{U}\lambda_{W}T_{W}\beta_{U}I_{0}\zeta_{W}^{-1}},$$

where $T_{U}={\left(\sigma_{U}+\mu_{I}+\phi_{I}I_{0}+\xi \right)}^{-1}$ and $T_{W}={\left(\sigma_{W}+\mu_{M}+\phi_{M}M_{0}\right)}^{-1}$. The value $R_{or}=1$ is the invasion threshold for *M. orientalis* in uninfected mussel populations. Where the host population is infected with *M. intestinalis* at steady state, the threshold is $R_{io}=1$. The expression for $R_{io}$ is identical to that for $R_{or}$, except that $I_{0}$ and $M_{0}$ are replaced with the steady-state population densities of immature and mature mussels, respectively, in the presence of *M. intestinalis*.

### 2.2. Derivation of parameter values

The parametrization of the model was based on a combination of a search of pre-existing literature, the results from our own experiments to quantify the relationship between key parameter values and temperature, and the collection of field data from the southern Wadden Sea in 2022–2024. Here, we briefly describe the sources of each parameter value in tables 1–3; for more details, see electronic supplementary material, tables S1–S3. The details of temperature experiments with the two parasites and mussels can be found in [14,18]. The host birth rate ν included the whole process from spawning until reaching a settled juvenile size that can get infected. Therefore, the estimate was derived from reported differences in mussel settlement success in the Wadden Sea based on winter temperatures that affect the presence of mussel predators [22]. Mussel mortality rates $\mu_{I}$ and $\mu_{M}$ were based on rates reported in [23], with a 10% increase in mortality at the higher temperature range as reported in [24]. The competition coefficients $\phi_{I}$ and $\phi_{M}$, that increase mortality in our model, included all competitive interactions between hosts. The values for these parameters were approximated based on two field studies [25,26]. To keep the model simple, we decided not to add a threshold level above which competition starts, but to lower the estimate of density-dependent increased mortality derived from [26] by one order of magnitude. Because immature mussels are more affected by predation than mature mussels [27], they are more likely to benefit from the predation protection provided by other mussels. Hence, the competition coefficient of immature mussels was chosen to be half that of mature mussels. The host maturation rate ξ was based on results reported in [28]. The effects of temperature on the growth of mussels were obtained from [29]. The four transmission rates from hosts (λ values) were based on a combination of the number of eggs per parasite [30,31], an estimate that approximately 30% of all adult parasites are females co-inhabiting an intestine with a male, and on development rates derived from our experiments [14,32]. The loss rates of free-living stages (ζ values) were also based on results from [14,32]. The transmission rates to hosts (β values) could not be derived from field data or experiments, because we could not determine the concentration of free-living stages in water in our system. Therefore, these parameters at the lower (current) temperature range were estimated by balancing the model so that the β values were consistent with field observations of the order of magnitude of parasite burdens. At the lower temperature range, most parasite loads in the field are in the range 0–10 copepods/host (unpublished field data of 1151 mussel dissections from Dutch Wadden Sea 2022–2024, electronic supplementary material, figure S1).

It was assumed that the ratio of β values of immature and mature mussels should be the same as the ratio of their filtration rates [33,34], because these intestinal parasites most likely enter their hosts with food. The change in β values by temperature was determined from the change in host filtration rates [33,34]. The rates of parasite mortality within hosts (σ values) were estimated from our experimental data [14,32]. The parasite load distribution from field data was also used to choose the simplest accurate shapes for parasite effect functions, $f(P_{U})$ and $g(P_{S})$, (electronic supplementary material, figure S1, see tables 2 and 3), along with the observation that in our experiment a single parasite caused a host effect with little increase in effect by each additional parasite [18]. There is insufficient knowledge on the biology of the processes involved on which to base a derivation of a suitable function, and we hence chose simple functions that at least mimic part of the intended feedback. These functions were assumed not to change with temperature and to be the same for all scenarios. In our experiment, the effect of $f(P_{U})$, relevant for *M. orientalis*, did not change by temperature, and even though the effect of $g(P_{S})$, relevant for *M. intestinalis*, was clearest at the lower range of temperatures, an earlier experiment had shown a similar effect at a higher temperature [35].

## 3. Results

At $10^{\circ }\text{C}$ and low values of the maturation rate of mussels, ξ, the basic reproduction number of both parasite species is below one, see table 4. At moderate and high values of ξ both parasite species have $R_{0}>1$, but *M. intestinalis* outcompetes *M. orientalis*: $R_{in}>R_{or}$. This means that when *M. intestinalis* is present, *M. orientalis* can only be maintained in the mussel population if there is immigration of parasite free-living stages from adjacent oyster populations. When *M. intestinalis* is not present, this immigration is not necessary for *M. orientalis* to persist in the mussel host.

**Table 4.** Basic reproduction numbers $R_{0}$ of the two parasite species at different values of the water temperature and rates of host maturation ξ. Values are $R_{in}$ : $R_{0}$ for *M. intestinalis*; $R_{or}$ : $R_{0}$ for *M. orientalis*; $R_{io}$ : if $R_{in}>1$ then $R_{0}$ for *M. orientalis* when *M. intestinalis* is at steady state, otherwise $R_{io}=R_{or}$.

at $10^{\circ }\text{C}$	$R_{in}$	$R_{or}$	$R_{io}$
$\xi =0.0014\;{\text{d}}^{-1}$	0.867	0.860	0.860
$\xi =0.0025\;{\text{d}}^{-1}$	1.014	1.007	0.988
$\xi =0.0030\;{\text{d}}^{-1}$	1.054	1.048	0.971

at $15^{\circ }\text{C}$	$R_{in}$	$R_{or}$	$R_{io}$
$\xi =0.0014\;{\text{d}}^{-1}$	0.651	0.803	0.803
$\xi =0.0025\;{\text{d}}^{-1}$	0.832	1.025	1.025
$\xi =0.0030\;{\text{d}}^{-1}$	0.880	1.084	1.084

**Figure 2.**
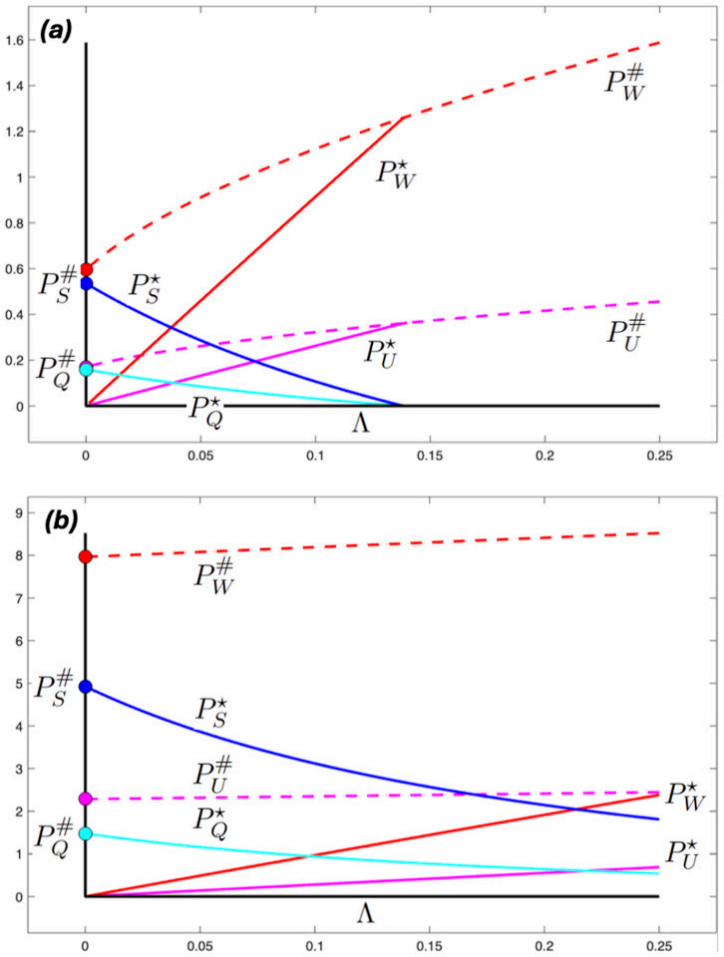
Model-derived steady states of the parasite populations at $10^{\circ }\text{C}$ as functions of Λ, the rate of immigration of *M. orientalis*. Magenta $\left(P_{U}\right)$ and red $\left(P_{W}\right)$: mean numbers of *M. orientalis* in immature and mature mussels, respectively; cyan $\left(P_{Q}\right)$ and blue $\left(P_{S}\right)$: mean numbers of *M. intestinalis* in immature and mature mussels, respectively. Broken lines show *M. orientalis* values in the absence of *M. intestinalis*. Filled circles on the vertical axis show *M. intestinalis* values in the absence of *M. orientalis*. Parameter values as in tables 1–3 with (*a*) $\xi =0.0025\;{\text{d}}^{-1}$, (*b*) $\xi =0.0030\;{\text{d}}^{-1}$.

In figure 2, parasite steady states are shown for two values of ξ and increasing values of the rate of immigration of *M. orientalis*, Λ. When $\xi =0.0025\;{\text{d}}^{-1}$ the two parasite species coexist in the mussel populations for $\Lambda <0.14\;\ell .\;{\text{d}}^{-1}$, but for higher rates of immigration, infection with *M. orientalis* reduces the size of the mussel population to a level where *M. intestinalis* is no longer supported. The parasite population dynamics are sensitive to the mussel population densities, going from Λ=0 to $\Lambda =0.14\;\ell .\;{\text{d}}^{-1}$ only changes the steady-state values of I and M by less than 3% (result not shown). In contrast, when $\xi =0.003\;{\text{d}}^{-1}$ both parasite species persist over the range of Λ considered, see figure 2.

**Figure 3.**
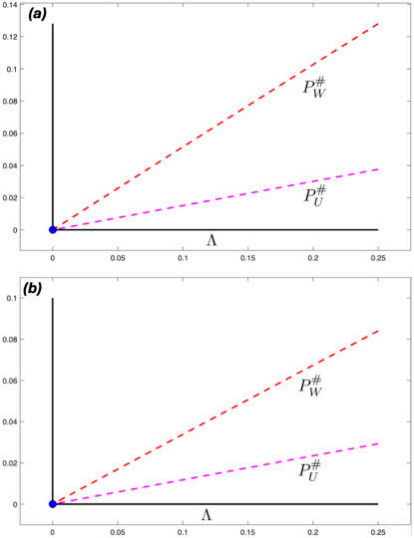
As in figure 2, but here the values of the steady states of the parasite populations are calculated when $\xi =0.0014\;{\text{d}}^{-1}$ again as functions of Λ, the rate of immigration of *M. orientalis*. Magenta $\left(P_{U}\right)$ and red $\left(P_{W}\right)$: mean numbers of *M. orientalis* in immature and mature mussels, respectively. Parameter values as in tables 1–3 with (*a*) temperature $10^{\circ }\text{C}$, (*b*) $15^{\circ }\text{C}$.

When $\xi =0.0014\;{\text{d}}^{-1}$, the lowest value considered, and at both temperatures considered, $R_{in}<1$ and *M. intestinalis* cannot persist in the mussel population. Even though $R_{or}<1$, *M. orientalis* can persist at steady state due to parasite immigration, Λ>0. These results are illustrated in figure 3. When the two higher values of ξ were considered at $15^{\circ }\text{C}$, we had $R_{in}<1$ and $R_{or}>1$, see table 4. Very high steady-state values of *M. orientalis* were obtained, with *M. intestinalis* failing to persist (see electronic supplementary material, figure S2).

**Figure 4.**
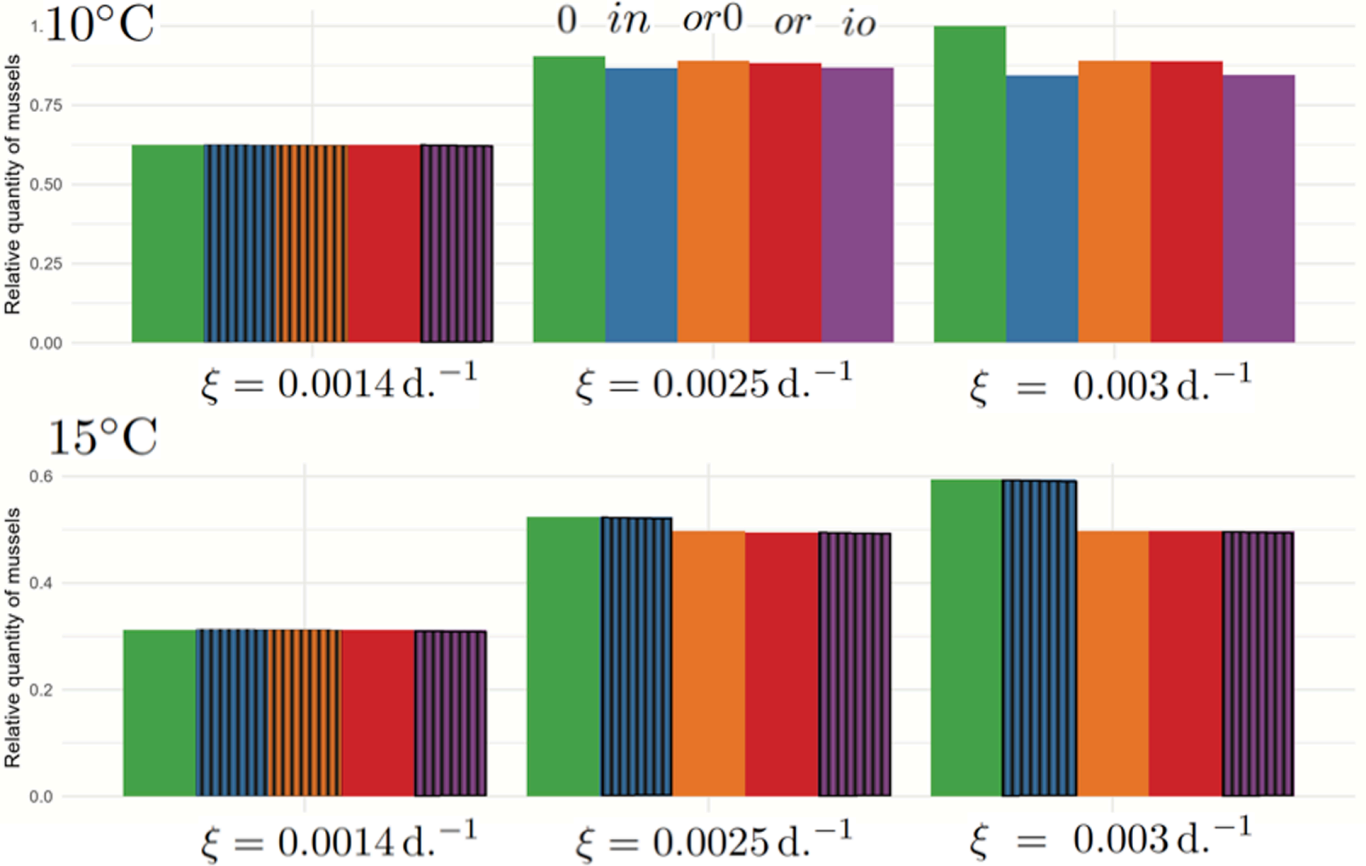
On the vertical axis are the values for the mussel steady states (I+M, total immature and mature) at indicated water temperatures and maturity rates ξ, relative to the baseline value of an uninfected population at $10^{\circ }\text{C}$ and $\xi =0.003\;{\text{d}}^{-1}$. Values are `0' (green): infection free; `in' (blue): with *M. intestinalis* only present in the population; `or0' (orange): with *M. orientalis* only and Λ=0; `or' (red): with *M. orientalis* only and $\Lambda =0.25\;\ell .\;{\text{d}}^{-1}$; `io' (purple): with both parasites present in the population and $\Lambda =0.25\;\ell .\;{\text{d}}^{-1}$. A striped entry indicates $R_{in}<1$, $R_{or}<1$, or both as appropriate, meaning that the parasite is not present in the steady state and thus does not affect the mussel population.

Figures 2 and 3 present results at different values of ξ, the maturation rate of mussels uninfected with *M. orientalis*. The effects of maturation rate, parasite infection and water temperature on mussel populations are shown in figure 4. The figure shows that increasing the water temperature to $15^{\circ }\text{C}$ reduces the mussel population density to between 50 and 60% of its baseline value at $10^{\circ }\text{C}$. At both temperatures, the lowest maturity rate corresponds to a much lower host population density, and infection with either or both parasite species only reduces the mussel population density by between 5 and 17% at the two highest maturation rates. When *M. intestinalis* is present, the addition of *M. orientalis* does not cause a further reduction in mussel population, and at $15^{\circ }\text{C}$, *M. intestinalis* is not maintained in the population. Going from Λ=0 to ℓ only changes the range of ξ at which *M. orientalis* is present, not its effect on hosts.

## 4. Discussion

In our model, a moderate temperature increase changed the maintenance communities of two closely related parasites in qualitatively different ways. In [1], it was shown that the concepts of maintenance host, maintenance community and reservoir (as proposed in [8]) depend on the ecosystem context of the species that are considered. The same species can be a maintenance host or a reservoir for a parasite in one context, but that capacity can be lost due to changes in one or more non-host species in the ecosystem. In the present analysis, we see that even without considering the wider ecosystem context, the interactions of two parasites with a single host species are important in determining whether that species plays a role in maintenance [1]. Not only direct changes in interacting species, but also abiotic factors that affect the communities change the populations required for parasite persistence. Another factor that affected the ability of a parasite to be maintained in a host species was the presence of another parasite: at the lower temperature, the second parasite required the presence of another host species to act as a reservoir when the first parasite was present, whereas without the first parasite mussels sufficed as a maintenance community (solid red and magenta lines start at zero while broken lines start at non-zero values when immigration of *M. orientalis* is zero in figure 2). This means that the presence of the first parasite provided resistance against the invasion of the second parasite.

**Figure 5.**
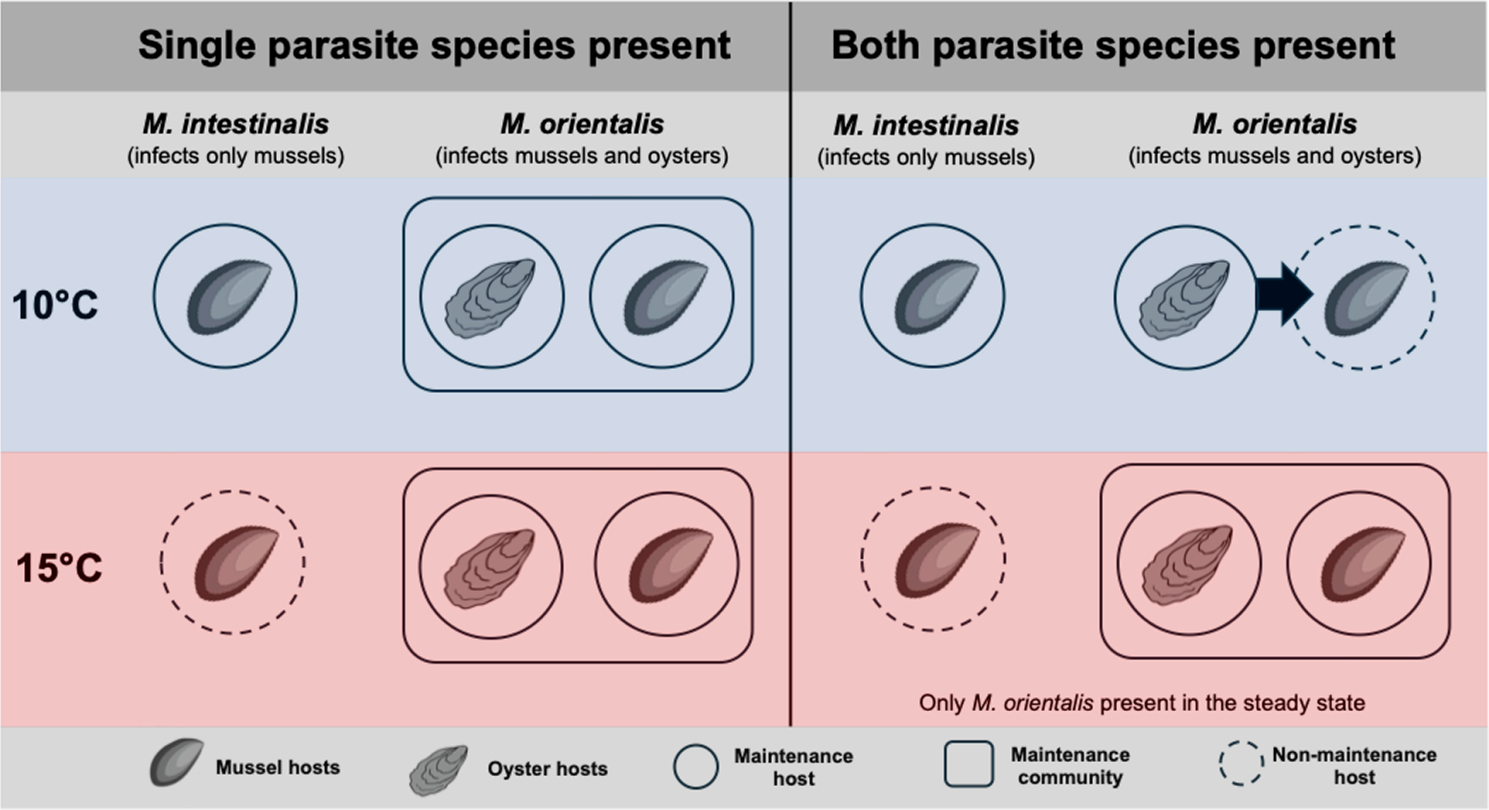
Graphic summary of our results on the maintenance of *M. intestinalis* and *M. orientalis* at the two temperature scenarios. The terminology for maintenance is based on Haydon *et al.* [8], see also [1]. We depict the situation in which *M. orientalis* larval immigration from oysters is always present and fixed at a low rate ($\Lambda =0.03\;{\text{d}}^{-1}$), and the mussel maturation rate is moderate to high ($0.0025\;{\text{d}}^{-1}<\xi <0.003\;{\text{d}}^{-1}$). We focus on the following situations: whether one or both parasite species are present and maintained and how this differs by temperature (10 and $15^{\circ }\text{C}$). Mussels are a maintenance host for *M. intestinalis* at $10^{\circ }\text{C}$, but not at $15^{\circ }\text{C}$, either alone or in the presence of *M. orientalis* (first and third columns). The maintenance community of *M. orientalis* depends on temperature and whether *M. intestinalis* is present. Mussels and oysters are a maintenance community for *M. orientalis* at both temperatures when *M. intestinalis* is absent (second column). When *M. intestinalis* is present (fourth column), at $10^{\circ }\text{C}$ larval immigration from oysters (arrow) is needed to sustain *M. orientalis* in mussels (hence without oysters, *M. orientalis* is not maintained in the system). At $15^{\circ }\text{C}$, mussels and oysters are a maintenance community for *M. orientalis*. In addition, the model results suggest that as a single species, mussels are a maintenance host for *M. orientalis* at $15^{\circ }\text{C}$ (i.e. without the influx of larvae from an oyster population).

Our model suggests that due to an increase in temperature within the range predicted by year 2100 and with other factors remaining constant, the established specialist parasite *M. intestinalis* will lose its ability to persist in the local mussel population, whereas the invading generalist parasite *M. orientalis* will gain the ability to persist. For that persistence, the original host, the Pacific oyster, is no longer necessary in this system. This transformation is illustrated in figure 5.

We also showed that adding a second invasive parasite species, one that is harmful to the hosts on the individual level, does not necessarily increase harm on the host population. When *M. intestinalis* was already present (blue bars in figure 4), the arrival of *M. orientalis* at the current temperature did not increase harm on the mussel population (purple bars in figure 4), but rather the population density trivially increased (both purple bars are 0.2% higher than the blue bars) when the mussel maturation rate was moderate to high ($0.0025\;{\text{d}}^{-1}<\xi <0.003\;{\text{d}}^{-1}$), corresponding to 333–400 days from settlement to spawning which is realistic for the Wadden Sea [28]. If *M. orientalis* was the only parasite in the system, it did decrease the host population (green versus orange and red bars in figure 4), an effect that seems slightly stronger at the elevated temperature. This is reflected in the positive trend in the mussel population observed since the arrival of *M. orientalis* in the Wadden Sea [36]. This finding fits with the theory that adding biodiversity can balance systems [37], even if the added species are not native. A facilitation between invasive species did occur in the form of oysters enabling the maintenance of *M. orientalis* at the lower temperature. Still, the effect of this facilitation on the population of native hosts was positive, and oysters could be seen to offer an ecosystem service for mussels by enabling the second parasite to be present. This effect became detrimental only at the higher temperature when *M. orientalis* became the only parasitic copepod in the system, which was also when oysters were no longer required to maintain their persistence.

This observation adds to the list of benefits mussels gain from Pacific oysters, despite them being an invasive species. Oysters have previously been shown to protect mussels from predation, storms and ice scouring [38,39]. The role of oysters is even more complex, because they also filter out larvae of both themselves and mussels [40] and they compete with mussels for food [38]. In this simplified model, oysters were included only as a source for infectious stages of *M. orientalis*. It would be interesting to add oysters as a full entity in the model. Our model is already complex, and adding all oyster interactions would have risked losing the insight from the simpler model.

In our model, no direct competition between the two parasites was added, so the negative effect *M. orientalis* had on *M. intestinalis* at the lower temperature occurred solely through host effects. In the northern Wadden Sea, *M. intestinalis* prevalence has decreased since the arrival of oysters with *M. orientalis*, and that effect has been attributed to *M. intestinalis* avoiding mussels already infected by *M. orientalis*, while *M. orientalis* tends to aggregate in already-infected mussels [19]. The decline in *M. intestinalis* numbers could also be because of a dilution effect by oysters that are not competent hosts but filter high volumes of water, as they have been previously shown to protect mussels from trematode infections by filtering out the infectious stages [41]. Both the avoidance of hosts already infected with *M. orientalis* and the dilution effect by oysters may play a role in the decline of *M. intestinalis*, but our model shows that the minor host effect due to *M. orientalis* alone suffices to explain a decline in *M. intestinalis* numbers.

We assumed that oysters suffice for the maintenance of *M. orientalis* at the pre-climate-change temperature. This implies the assumption that their critical community size is high enough to keep $R_{or}>1$. Based on the genetic evidence of multiple introductions of *M. orientalis* from the native range, where it infects oysters, and the first detection of *M. orientalis* typically coinciding or taking place after the invasion of oysters [15–17], it seems plausible that already-low initial populations of introduced oysters are able to maintain the parasite. In the current Wadden Sea, oysters dominate most intertidal reefs and are not likely to suffer from the predicted increase in temperature, because the temperature in their original range is already higher than that predicted for the Wadden Sea by 2100 [39,42].

When considering experimental data on temperature effects on parasite life cycles, the two parasites appear to be strikingly similar, and the number of life cycles they can complete at different temperature scenarios differs little [14]. The main difference in the parameter values of the two parasites was the poorer adaptation of *M. orientalis* for low temperatures, which is logical considering how water temperature rarely goes below $13^{\circ }\text{C}$ in the parasite’s original range [14,42]. In our model, including data on host effects and other host species changed the outcome of temperature increase on the two parasites, indicating that very simplified predictions for one species can be misleading if interacting species in the ecosystem are ignored.

Adaptation to the changing environment through evolution in the next 75 years was not included in the model. This was to enable the analysis of the already complex model and because of uncertainty in the speed of warming. The annual mean water temperature in the southern Wadden Sea has stayed above $12.6^{\circ }\text{C}$ for 2022–2024, which is $2.4^{\circ }\text{C}$ higher than the annual mean of 1900–1999 and $1.0^{\circ }\text{C}$ higher than that of 2000–2020, making it possible that the existing predictions severely underestimate the speed of warming [43]. Furthermore, the warming might not be spread evenly across the seasons in this region but rather be more pronounced in the summer [44]. These factors can limit the ability of adaptation to keep up, while evolution still increases the uncertainty of our predictions. The adaptive capacity of the two parasite species is likely to differ because of their large differences in genetic diversity; the chances for *M. intestinalis* to adapt to the fast change in temperature do not appear promising due to it having passed through a genetic bottleneck on its invasion pathway. In contrast, the high genetic diversity and environmental tolerance of Wadden Sea *M. orientalis* makes it more likely to adapt to the changing environment [15,16]. The maintenance of *M. intestinalis* may in future instead be facilitated by distributional shifts of a congeneric species, the southern Mediterranean mussel (*Mytilus galloprovincialis*), which is adapted to higher temperatures, making it possible that the blue mussels of the southern Wadden Sea will be replaced and hybridized with this species rather than merely decline [45]. The drop in the critical community size of *M. edulis* to below the threshold required for the maintenance of *M. intestinalis* is less likely to happen with temperature increase if the species is hybridized with or replaced by *M. galloprovincialis*. The continued presence of *M. intestinalis* in *M. galloprovincialis* populations in southern sites of the European Atlantic decades after the first occurrence of *M. intestinalis* supports this possibility [15]. This underlines the possibility that species increasing their range with or without the help of humans is not necessarily detrimental but can also increase the resilience of ecosystems.

Our model has 20 parameters, 17 of which change with temperature. All parameter values, except the transmission rates to hosts, β, were based on best available information for the system, and extensive effort was put into getting them as accurate as possible. Only the final mortality rate of free-living *M. orientalis* free-living stages, $\zeta_{W}$ at $15^{\circ }\text{C}$ ($\zeta_{W}=0.3\;{\text{d}}^{-1}$), differed from the value derived from experimental results. This was for two reasons: when we analysed the model with the value based only on larval lifespan in the experimental data at $15^{\circ }\text{C}$ ($\zeta_{W}=0.2\;{\text{d}}^{-1}$), *M. orientalis* loads per mussel were in the hundreds when $0.0025\;{\text{d}}^{-1}<\xi <0.003\;{\text{d}}^{-1}$, which is not realistic for this parasite based on field data [15,19]. This value also resulted in slightly higher effect on host populations at $15^{\circ }\text{C}$, but it did not affect any other outcomes of our model. The second reason was that in real ecosystems, the mortality of free-living stages is determined by their innate lifespan, predation, getting washed away by tides and salinity [16,46], and temperature increase is likely to increase predation [46]. Increasing the mortality rate of *M. intestinalis* free-living stages at $15^{\circ }\text{C}$ to reflect this increase in predation did not affect the model outcome, as already the low estimate resulted in elimination of the parasite population, but increasing the mortality rate of *M. orientalis* free-living stages increased the realism of the model output. The final ζ values still reflect the longer lifespan of *M. orientalis* free-living stages at $15^{\circ }\text{C}$ compared with $10^{\circ }\text{C}$, and compared with *M. intestinalis* free-living stages at $15^{\circ }\text{C}$, as seen in the experiment [32]. The effect of temperature change on mussel maturation is not straightforward, because even though temperature may boost growth by increasing ectotherm metabolic rates when no food limitation is present [47], food availability is affected by several environmental variables, including temperature and even the location of mussels in a mixed oyster–mussel reef [38,48]. Also, the genetics of the particular mussel population can modify the effect of environmental variables on growth [29]. For this reason, we decided to analyse the model with three levels of mussel maturation at both temperatures, the most appropriate range being determined by the system and situation of interest.

In our conceptual approach, we have ignored many dimensions of heterogeneity in the real system influencing the mussel–oyster–parasite interaction that we have studied in isolation. Hence, our aim is decidedly not to predict the time evolution of population abundance of mussels and their parasites. Our aims and conclusions are more qualitative and potentially more robust than predicting spatio-temporal variation in detail. In our isolated system, everything is kept constant apart from our parameters of interest, and many aspects are not considered at all. For example, we have assumed that the temperatures are constant, whereas in reality they vary with time (over the days, weeks and months) and with space at any given moment (with shallow parts experiencing different regimes and extremes). On top of that, there are many factors that we ignore such as spatial heterogeneity, tidal movement, predators, currents, food dynamics and competitors. One important competitor of the mussel is the oyster. We have also chosen to include the oyster population as a constant and have explored the influence of oyster population through its smaller or larger (constant) production of infectious stages of the *M. orientalis*. This means that we have ignored the population dynamics of oysters and any other feedback to and from the mussel population (for example, through competition for food, but also through providing shelter for mussels against their predators). In addition, we only consider the potential effect of a long-term rise in temperature, whereas there are other effects of climate change in systems like the Wadden Sea that may be relevant and impact the mussel–oyster–parasite system. One can think of sea level rise, changes in salinity, future invasions and abundance, composition and distributional changes in the ecosystem (see for example [49]).

In our system of interest, a small change in the average environmental temperature sufficed to cause a change in the maintenance communities and host population effects of two invasive parasites. Mussels did not act as maintenance hosts for *M. orientalis* at the pre-climate-change temperatures of the Wadden Sea because of the parasite’s poor adaptation for cold temperature and the presence of another parasite, *M. intestinalis*. This adaptation for higher temperatures proved beneficial in the higher temperature range predicted to occur due to climate change by 2100, and being a generalist that had invaded the system with its original host enabled *M. orientalis* to persist also at the lower temperature. The future of the specialist *M. intestinalis* in this system appears less promising because of its dependence on one host species that is predicted to suffer from the increase in temperature. These findings highlight how small changes in the environment can change parasite persistence and host effects, and the importance of including the environmental change effects of not only the species of interest but also the species it interacts with in predictions.

## Data Availability

Available from: [50]. Supplementary material is available online [51].

## Authors' Notes

Incorrect initials affects "Author contributions"

ERJ: conceptualization, formal analysis, investigation, methodology, validation, visualization, writing - original draft and editing; AvL: conceptualization, writing - review and editing; KMW: conceptualization, writing - review and editing; DWT: conceptualization, funding acquisition, project administration, supervision, writing - review and editing; JAPH: funding acquisition, project administration, supervision, conceptualization, investigation, methodology, writing-original draft, and editing; MGR: conceptualization, formal analysis, investigation, methodology, writing - original draft and editing;

## References

[1] Roberts MG, Heesterbeek JAP. 2020 Characterizing reservoirs of infection and the maintenance of pathogens in ecosystems. J. R. Soc. Interface **17**, 20190540. (10.1098/rsif.2019.0540) 31937232 PMC7014803

[2] Budria A, Candolin U. 2014 How does human-induced environmental change influence host-parasite interactions? Parasitology **141**, 462–474. (10.1017/s0031182013001881) 24477175

[3] Hellmann JJ, Byers JE, Bierwagen BG, Dukes JS. 2008 Five potential consequences of climate change for invasive species. Conserv. Biol. **22**, 534–543. (10.1111/j.1523-1739.2008.00951.x) 18577082

[4] Dobson AP, Hudson PJ. 1992 Regulation and stability of a free-living host-parasite system: Trichostrongylus tenuis in red grouse. II. Population models. J. Anim. Ecol. **61**, 487–498. (10.2307/5339)

[5] Tompkins DM *et al*. 2002 Parasites and host population dynamics. In The ecology of wildlife diseases (eds PJ Hudson, A Rizzoli, BT Grenfell, JAP Heesterbeek, AP Dobson), pp. 45–62. Oxford, UK: Oxford University Press. (10.1093/oso/9780198506201.003.0003)

[6] Sures B, Nachev M, Schwelm J, Grabner D, Selbach C. 2023 Environmental parasitology: stressor effects on aquatic parasites. Trends Parasitol. **39**, 461–474. (10.1016/j.pt.2023.03.005) 37061443

[7] Johnson PTJ, de Roode JC, Fenton A. 2015 Why infectious disease research needs community ecology. Science **349**, 1259504. (10.1126/science.1259504) 26339035 PMC4863701

[8] Haydon DT, Cleaveland S, Taylor LH, Laurenson MK. 2002 Identifying reservoirs of infection: a conceptual and practical challenge. Emerg. Infect. Dis. **8**, 1468–1473. (10.3201/eid0812.010317) 12498665 PMC2738515

[9] Cardeccia A, Marchini A, Occhipinti-Ambrogi A, Galil B, Gollasch S, Minchin D, Narščius A, Olenin S, Ojaveer H. 2018 Assessing biological invasions in European seas: biological traits of the most widespread non-indigenous species. Estuar. Coast. Shelf Sci. **201**, 17–28. (10.1016/j.ecss.2016.02.014)

[10] Goedknegt MA, Feis ME, Wegner KM, Luttikhuizen PC, Buschbaum C, Camphuysen KCJ, van der Meer J, Thieltges DW. 2016 Parasites and marine invasions: ecological and evolutionary perspectives. J. Sea Res. **113**, 11–27. (10.1016/j.seares.2015.12.003)

[11] Dunn AM *et al*. 2012 Indirect effects of parasites in invasions. Funct. Ecol. **26**, 1262–1274. (10.1111/j.1365-2435.2012.02041.x)

[12] Thieltges DW *et al*. 2025 Integrating climate change, biological invasions, and infectious wildlife diseases. Front. Ecol. Environ. **23**, e2849. (10.1002/fee.2849)

[13] van Aken HM. 2008 Variability of the water temperature in the western Wadden Sea on tidal to centennial time scales. J. Sea Res. **60**, 227–234. (10.1016/j.seares.2008.09.001)

[14] Jolma ERE, Born-Torrijos A, Heesterbeek H, van Leeuwen A, van Leeuwen SM, Twijnstra RH, Wegner KM, Thieltges DW. 2024 Warming effects on the life cycles of two parasitic copepods with different invasion histories. Ecol. Evol. **14**, e11485. (10.1002/ece3.11485) 38932946 PMC11199328

[15] Feis ME *et al*. 2019 Global invasion genetics of two parasitic copepods infecting marine bivalves. Sci. Rep. **9**, 12730. (10.1038/s41598-019-48928-1) 31484951 PMC6726661

[16] Brenner M, Schulze JJ, Fischer J, Wegner M. 2019 First record of the parasitic copepod (Mytilicola orientalis Mori, 1935) in blue mussels (Mytilus spp.) of the Baltic Sea. BioInvasions Rec. **8**, 623–632. (10.3391/bir.2019.8.3.19)

[17] Ewers-Saucedo C, Heuer N, Moesges Z, Ovenbeck K, Schröter N, Brandis D. 2020 First record of the Pacific oyster Magallana gigas (Thunberg, 1793) in the Baltic Sea proper. Mar. Biodivers. Rec. **13**, 9. (10.1186/s41200-020-00193-2)

[18] Jolma ER, Born-Torrijos A, Engelsma MY, Heesterbeek H, van Leeuwen A, Twijnstra RH, Wegner KM, Thieltges DW. 2025 Temperature effects on the impact of two invasive parasitic copepods on the survival, growth, condition, and reproduction of native mussels. Biol. Invasions **27**, 67. (10.1007/s10530-024-03527-8)

[19] Feis ME, Gottschalck L, Ruf LC, Theising F, Demann F, Wegner KM. 2022 Invading the occupied niche: how a parasitic copepod of introduced oysters can expel a congener from native mussels. Front. Mar. Sci. **9**. (10.3389/fmars.2022.915841)

[20] Diekmann O, Heesterbeek JAP, Britton T. 2013 Mathematical tools for understanding infectious disease dynamics. Princeton, NJ: Princeton University Press.

[21] MatLab 2025a. 2025 The MathWorks Inc.

[22] Beukema J, Dekker R. 2014 Variability in predator abundance links winter temperatures and bivalve recruitment: correlative evidence from long-term data in a tidal flat. Mar. Ecol. Prog. Ser. **513**, 1–15. (10.3354/meps10978)

[23] McGrorty S, Clarke R, Reading C, Goss-Custard J. 1990 Population dynamics of the mussel Mytilus edulis: density changes and regulation of the population in the Exe estuary, Devon. Mar. Ecol. Prog. Ser. **67**, 157–169. (10.3354/meps067157)

[24] Seuront L, Nicastro KR, Zardi GI, Goberville E. 2019 Decreased thermal tolerance under recurrent heat stress conditions explains summer mass mortality of the blue mussel Mytilus edulis. Sci. Rep. **9**, 17498. (10.1038/s41598-019-53580-w) 31767954 PMC6877631

[25] McGrorty S, Goss-Custard J. 1995 Population dynamics of Mytilus edulis along environmental gradients:density-dependent changes in adult mussel numbers. Mar. Ecol. Prog. Ser. **129**, 197–213. (10.3354/meps129197)

[26] van de Koppel J, Rietkerk M, Dankers N, Herman PMJ. 2005 Scale-dependent feedback and regular spatial patterns in young mussel beds. Am. Nat. **165**, E66–77. (10.1086/428362) 15729660

[27] Seed R, Suchanek T. 1992 Population and community ecology of *Mytilus*. In The mussel Mytilus: ecology, physiology, genetics and culture (ed. EM Gosling), pp. 87–169. Amsterdam, The Netherlands: Elsevier.

[28] Sprung M. 1983 Reproduction and fecundity of the mussel Mytilus edulis at Helgoland (North sea). Helgolander Meeresunters. **36**, 243–255. (10.1007/BF01983629)

[29] Kamermans P, Saurel C. 2022 Interacting climate change effects on mussels (Mytilus edulis and M. galloprovincialis) and oysters (Crassostrea gigas and Ostrea edulis): experiments for bivalve individual growth models. Aquat. Living Resour. **35**, 1. (10.1051/alr/2022001)

[30] Korringa P. 1968 On the ecology and distribution of the parasitic copepod Mytilicola intestinalis. Steuer. Bijdr. Tot. Dierkd. **38**, 47–57. (10.1163/26660644-03801008)

[31] Johnson EA, Chew KK. 1969 Preliminary report on the fecundity of Mytilicola orientalis. J. Fish. Res. Board Can. **26**, 2245–2246. (10.1139/f69-214)

[32] Jolma ER. 2024 Data and code for warming effects on the life cycles of two parasitic copepods with different invasion histories. NIOZ. (10.25850/nioz/7b.b.4g) PMC11199328 38932946

[33] Riisgard HU. 1991 Filtration rate in the blue mussel Mytilus edulis Linnaeus, 1758: dependence on algal concentration. J. Shellfish Res. **10**, 29–35.

[34] Kittner C, Riisgård H. 2005 Effect of temperature on filtration rate in the mussel Mytilus edulis: no evidence for temperature compensation. Mar. Ecol. Prog. Ser. **305**, 147–152. (10.3354/meps305147)

[35] Feis ME, Goedknegt MA, Thieltges DW, Buschbaum C, Wegner KM. 2016 Biological invasions and host–parasite coevolution: different coevolutionary trajectories along separate parasite invasion fronts. Zoology **119**, 366–374. (10.1016/j.zool.2016.05.012) 27373339

[36] Wageningen University and Research (WUR). 2020 Schelpdiermonitor. See https://www.wur.nl/nl/artikel/schelpdiermonitor.htm (accessed 10 December 2024).

[37] Keesing F *et al*. 2010 Impacts of biodiversity on the emergence and transmission of infectious diseases. Nature **468**, 647–652. (10.1038/nature09575) 21124449 PMC7094913

[38] Eschweiler N, Christensen HT. 2011 Trade-off between increased survival and reduced growth for blue mussels living on Pacific oyster reefs. J. Exp. Mar. Biol. Ecol. **403**, 90–95. (10.1016/j.jembe.2011.04.010)

[39] van der Meer J, Dankers N, Ens BJ, van Stralen M, Troost K, Waser AM. 2019 The birth, growth and death of intertidal soft-sediment bivalve beds: no need for large-scale restoration programs in the Dutch Wadden Sea. Ecosystems **22**, 1024–1034. (10.1007/s10021-018-0320-7)

[40] Troost K, Kamermans P, Wolff WJ. 2008 Larviphagy in native bivalves and an introduced oyster. J. Sea Res. **60**, 157–163. (10.1016/j.seares.2008.04.006)

[41] Thieltges DW, Reise K, Prinz K, Jensen KT. 2009 Invaders interfere with native parasite–host interactions. Biol. Invasions **11**, 1421–1429. (10.1007/s10530-008-9350-y)

[42] Japan Meteorological Agency. 2024 Sea surface temperature (around Japan). See https://www.data.jma.go.jp/gmd/kaiyou/english/long_term_sst_japan/ sea_surface_temperature_around_japan.html (accessed 16 December 2024).

[43] Royal Netherlands Institute for Sea Research. 2024 Sea water temperature. See https://www.nioz.nl/en/expertise/wadden-delta-research-centre/data-tools/long-term-ecological-time-series/sea-water-temperature (accessed 16 December 2024).

[44] de Winter NJ *et al*. 2024 Amplified seasonality in western Europe in a warmer world. Sci. Adv. **10**, eadl6717. (10.1126/sciadv.adl6717) 38748800 PMC11095466

[45] Fly EK, Hilbish TJ, Wethey DS, Rognstad RL. 2015 Physiology and biogeography: the response of European mussels (Mytilus spp.) to climate change. Am. Malacol. Bull. **33**, 136–149. (10.4003/006.033.0111)

[46] Goedknegt MA, Welsh JE, Drent J, Thieltges DW. 2015 Climate change and parasite transmission: how temperature affects parasite infectivity via predation on infective stages. Ecosphere **6**, 1–9. (10.1890/es15-00016.1)

[47] Gillooly JF, Brown JH, West GB, Savage VM, Charnov EL. 2001 Effects of size and temperature on metabolic rate. Science **293**, 2248–2251. (10.1126/science.1061967) 11567137

[48] Jacobs P, Kromkamp J, van Leeuwen S, Philippart C. 2020 Planktonic primary production in the western Dutch Wadden Sea. Mar. Ecol. Prog. Ser. **639**, 3–71. (10.3354/meps13267)

[49] Philippart C *et al*. 2024 Wadden Sea quality status report: climate change. Zenodo (10.5281/zenodo.15111640)

[50] Jolma R. 2024 Data and code for ‘Warming effects on the life cycles of two parasitic copepods with different invasion histories’. (10.25850/nioz/7b.b.4g) PMC11199328 38932946

[51] Jolma RJ, van Leeuwen A, Wegner KM, Thieltges D, Heesterbeek H, Roberts M. 2025 Supplementary material from: Context-dependency of maintenance communities of invasive parasites under climate change: a case study of mussels and intestinal copepods in the Wadden Sea. Figshare. (10.6084/m9.figshare.c.8078988) PMC12587053 41189498

